# miR-210 loss leads to widespread phenotypic and gene expression changes in human 293T cells

**DOI:** 10.3389/fgene.2024.1486252

**Published:** 2024-12-16

**Authors:** Xiaoxiao Zhang, Zhen Meng, Chengyong Yang, Chenghao Wang, Kexin Zhang, Anxin Shi, Jingjing Guo, Yong Feng, Yan Zeng

**Affiliations:** ^1^ Department of Zoology, College of Life Sciences, Nanjing Agricultural University, Nanjing, Jiangsu, China; ^2^ Centre in Artificial Intelligence Driven Drug Discovery, Faculty of Applied Sciences, Macao Polytechnic University, Macao, China; ^3^ Institute of Medical Virology, School of Basic Medical Sciences, Wuhan University, Wuhan, Hubei, China

**Keywords:** miR-210, CRISPR/Cas9, target gene, apoptosis, Bnip3L

## Abstract

**Introduction:**

Hypoxia responses are critical for myriad physiological and pathological processes, such as development, tissue repair, would healing, and tumorigenesis. microRNAs (miRNAs) are a class of small non-coding RNAs that exert their functions by inhibiting the expression of their target genes, and miR-210 is the miRNA universally and most conspicuously upregulated by hypoxia in mammalian systems. For its relationship to hypoxia, miR-210 has been studied extensively, yet no consensus exists on the roles and mechanisms of miR-210 in human physiological processes or diseases, and we know little about genuine miR-210 target genes in humans.

**Methods:**

To better investigate the functions and mechanisms of human miR-210, therefore, we derived the human *miR-210* gene knockout (KO) 293T cell lines using the CRISPR/Cas9 technology. We then examined the cellular phenotypes and gene expression profiles of 293T cells under normoxia and hypoxia conditions.

**Results and Discussion:**

We found that the loss of miR-210 altered a variety of cellular phenotypes including proliferation and apoptosis. Subsequent global gene expression analyses identified plausible mechanisms underlying these phenotypic changes in 293T cells. In particular, we showed that miR-210 might target the expression of BNIP3L as a potential mechanism to suppress apoptosis. Surprisingly, the mRNA levels of most previously reported miR-210 target genes were not induced upon *miR-210* KO, suggesting a need to reexamining and studying human miR-210 functions directly and comprehensively. Thus, our work established a human cellular system and opportunity to unravel the complexity of the regulatory networks by miR-210.

## Introduction

miRNAs are a large family of approximately 22 nucleotides long RNA molecules that play important regulatory roles in both physiological processes and diseases such as cancers (Bartel, 2018; Nair et al., 2014). miR-210-3p (referred to as miR-210 hereafter) is a miRNA widely expressed in mammalian tissues and universally induced by hypoxic conditions (Kulshreshtha et al., 2007; Khalilian et al., 2023). Such induction is due to the presence of binding sites for hypoxia-inducible factors (HIFs), such as HIF-1α, on the promoter of the *miR-210* gene. HIF-1α is the master regulatory protein in hypoxia responses: under hypoxic conditions, its stability significantly increases, allowing it to function effectively as a transcription factor to regulate the transcription of downstream genes (Kaelin and Ratcliffe, 2008). These downstream genes then mediate a series of responses to hypoxia, which are crucial for the metabolism and physiology of organisms, and biological processes such as embryonic organ development and formation, heart function, blood clotting, tissue repair, cell and tumor migration. For instance, tumor cells are often situated in low oxygen environments, and their survival, proliferation, and metastasis depend on their ability to overcome the adverse effects of hypoxia (Hompland et al., 2021).

Animal miRNAs bind to complementary sequences in the target mRNAs to suppress their expression (Bartel, 2018). Given the potential relationship between miR-210 and hypoxia, extensive research has focused on identifying miR-210 target genes, and activities and functions of those target genes would then point to the impact of miR-210 on various physiological phenomena. For instance, miR-210 might control development and signaling by inhibiting *EFNA3*, *HOXA1*, and *PTP1B* (Kuijper et al., 2007; Noman et al., 2012; Nakamura et al., 2008). miR-210 target genes associated with the cell cycle include *PLK1*, *CDC25B*, *CCNF*, *BUB1B*, *MNT*, and *FAM83D* (Zhang et al., 2009; He et al., 2013). Target genes like *E2F3*, *CASP8AP2*, *AIFM3*, *BNIP3*, and *SIN3A* could influence apoptosis (Ivan and Huang, 2014; Giannakakis et al., 2008; Wang et al., 2013; Shang et al., 2014). As hypoxia reduces mitochondrial respiration, miR-210 could regulate metabolism by suppressing the expression of the iron-sulfur cluster scaffold proteins *ISCU1/2* (Chan et al., 2009). The expression and function of miR-210 in tumors has also been a focus of research. miR-210 levels vary in many tumors, and anticancer therapies further alter miR-210 expression (Khalilian et al., 2023; Qin et al., 2014; Dang and Myers, 2015; Bavelloni et al., 2017; Feng et al., 2019; Balachandran et al., 2020; Camps et al., 2008; Foekens et al., 2008; Greither et al., 2010). Examples of cancers with elevated miR-210 levels include breast cancer, lung cancer, renal cell carcinoma, pancreatic cancer, osteosarcoma, while ovarian cancer, esophageal cancer, among others, exhibit decreased miR-210 levels. Malignant gliomas have reported both elevated and decreased miR-210 (Khalilian et al., 2023; Balachandran et al., 2020; Malzkorn et al., 2010; Wang J. et al., 2014; Lai et al., 2014; Lee et al., 2015). While miR-210 levels increase in renal cell carcinoma, inhibiting miR-210 levels could potentially worsen tumor progression (McCormick et al., 2013; Yoshino et al., 2017a). It is clear, therefore, that the relationship between miR-210 and disease severity is complicated.

Despite much effort, our understanding of miR-210 remains unsatisfactory, due to two main reasons. The first reason is that research on miR-210 has been highly fragmented, especially with regard to human miR-210. Published studies have mostly focused on distinct target genes, cellular behaviors, and diseases, with few overlaps or comprehensive analyses of miR-210 functions and targets. *In vivo* studies of miR-210 have been conducted in mice, yet *miR-210* KO mice are largely normal, with only mild phenotypes related to autoimmunity and pulmonary hypertension (Wang H. et al., 2014; Mok et al., 2013; White et al., 2015; Krawczynski et al., 2016; Bian et al., 2021; Watts et al., 2021), which do not correlate with the functions of most reported miR-210 target genes. Thus, it is possible that many miR-210 target genes identified thus far are not the genuine or the most physiologically relevant targets, or that miR-210 functions may differ between mice and humans. Moreover, hypoxia response and reported human miR-210 targets have not been examined systematically in cells lacking miR-210. The second reason is that the functions of miR-210 could be complex. While hypoxia activates miR-210, what miR-210 does or accomplishes afterwards might not be straightforward or immediately obvious. For example, are the overall miR-210 functions pro-hypoxia or anti-hypoxia responses? Considering that a single miRNA has hundreds of or even more target genes (Bartel, 2018), it is likely that different miR-210 target genes have opposing functions, with the ultimate biological outcomes dependent on the cell types, environments, and conditions. Therefore, there is a need to study human miR-210 specifically, directly and systematically, and to gain a more accurate understanding of miR-210 target genes and mechanisms in human systems. So in this study we constructed *miR-210* KO in 293T cells, a human cell line commonly used in mechanistic studies, assessed miR-210s impact on cell phenotypes and gene expression, and evaluated the potential mechanisms on human cells and hypoxia responses by miR-210.

## Materials and methods

### Molecular cloning

Restriction enzymes, PCR polymerases, and T4 DNA Ligase were from New England BioLabs (Ipswich, MA, United States). pRL-CMV, the control, Renilla luciferase expressing plasmid, was from Promega (Madison, WI, United States). The single guide RNA (sgRNA) targeting the human *miR-210* gene was designed using the website https://www.benchling.com/crispr/, and the corresponding oligos were 5′-CAC​CGA​GGG​GCT​GCC​CTG​CGC​CTG​G-3′ and 5′-AAA​CCC​AGG​CGC​AGG​GCA​GCC​CCT​C-3’. The oligos were synthesized by Sangon (Shanghai, China) and cloned into the BsmbI sites in LentiCRISPR (Sanjana et al., 2014) to obtain LentiCRISPR-miR-210, which was verified by Sanger sequencing (Sangon). Coding sequences of the full length BNIP3L (NM_004331) and its miR-210 binding site mutant were synthesized and cloned into the p3xFLAG-CMV 10 vector (General Biology, Anhui, China). To construct a firefly luciferase (luc) reporter with BNIP3L in the 3′ untranslated region, BNIP3L sequence including the putative miR-210 binding site was amplified from the above p3xFLAG-CMV-BNIP3L plasmid with primers 5′-GCG​CTA​GCG​GAA​AAT​GAG​CAG​TCT​CT-3′ and 5′-GCC​TCG​AGA​TTC​ATG​TTG​TGC​A-3′, digested with NheI and XhoI, and cloned into pCMV-luc (Ghosh et al., 2010). The BNIP3L mutant lucierase reporter with the miR-210 binding seed sequence 5′-ACACGTAC-3′ deleted was constructed using the Quikchange method (Stratagene, La Jolla, CA, USA) with primers 5′-AAA​TGG​GGG​GCT​GGA​CAT​CCT​CAT​CCT​CCA-3′ and 5′- TGG​AGG​ATG​AGG​ATG​TCC​AGC​CCC​CCA​TTT -3’. Plamsids were verified by Sanger sequencing.

### Cell cultures

The human 293T cell line was acquired from Sangon. Cells were cultured in the Dulbecco’s modified Eagle’s medium supplemented with 10% fetal bovine serum and 2 mM L-glutamine (Invitrogen, Waltham, MA, United States) and maintained at 37°C with 5% CO_2_ in a cell culture incubator. Cells were transfected with plasmid DNAs (and the control or miR-210 mimic RNA (Sangon), if necessary), with 400 ng of total nucleic acids per 24-well, using Lipofectamine 2000 (Invitrogen). For hypoxia testing, cells were placed in a modular incubator chamber (Billups-Rothenberg, San Diego, CA, United States) and flushed with 95% N_2_/5% CO_2_, which reduced the oxygen level to approximately 3% (Ghosh et al., 2010). Or more commonly, cells were treated with the control DMSO (Sangon) or 0.5 mM dimethyloxalylglycine (DMOG, catalog number 71210, Cayman Chemical, Ann Arbor, MI, USA) for 24 h, unless indicated otherwise.

### Construction of miR-210 KO 293T cell lines

LentiCRISPR-miR-210 was transfected into 293T cells, and the wild-type (WT) control transfected with the empty LentiCRISPR vector. After 48 h cells were transferred to 10 cm culture dishes (Sangon) and selected with 2 μg/mL puromycin (catalog number ST551, Beyotime, Shanghai, China). Single clones were picked using cloning rings (Corning, Corning, NY, United States), expanded, and their *miR-210* status determined by PCR and sequencing. For PCR, genomic DNA was isolated from 293T cells using the FastPure Cell/Tissue DNA Isolation Mini Kit (Vazyme, Nanjing, China), and amplified with primers 5′-GCA​AGC​TTC​GGG​GGG​TCG​GGC​T-3′ and 5′-GGG​TAT​CTG​GCC​CAG​CCT-3’. PCR products were inserted into the pMD19-T vector (Takara, Beijing, China), and approximately 10 isolates for each 293T clone examined by Sanger sequencing to identify the *miR-210* KO cells. To assess the off-target effects of the sgRNA, potential off-target sites were predicted using the website https://www.benchling.com/crispr/, primers designed (Supplementary Table S1) to amplify the genomic DNA, and the PCR products analyzed by Sanger sequencing.

### RNA isolation and real-time PCR (qPCR) analyses

Total RNA was isolated from 293T cells using Trizol (Invitrogen). For miR-210 expression analysis, the miRNA 1st Strand cDNA Synthesis Kit (Vazyme) was used to reverse transcribe RNA with the miR-210-specific primer 5′-GTC​GTA​TCC​AGT​GCA​GGG​TCC​GAG​GTA​TTC​GCA​CTG​GAT​ACG​AC-3’. For mRNA expression analyses, the SuperScript III First-Strand Synthesis SuperMix (Invitrogen) was used to reverse transcribe RNA. qPCR reactions were carried out on the QuantStudio 6 Flex Real-Time PCR System (Thermo Fisher Scientific, Waltham, MA, United States) using the SYBR Green qPCR Master Mix (Thermo Fisher Scientific). The relative expression of target genes was calculated with the 2^−ΔΔCT^ method normalized to that of the U6 or Actin mRNA (Zhang et al., 2013). qPCR primers were synthesized by Sangon: miR-210: 5′-CGC​TGT​GCG​TGT​GAC​AGC-3′ and 5′-AGT​GCA​GGG​TCC​GAG​GTA​TT-3′, U6: 5′-GCT​TCG​GCA​GCA​CAT​ATA​CTA​AAA​T-3′ and 5′-CGC​TTC​ACG​AAT​TTG​CGT​GTC​AT-3′, Actin: 5′-GGA​CTT​CGA​GCA​AGA​GAT​GG-3′ and 5′-AGC​ACT​GTG​TTG​GCG​TAC​AG-3′, E2F3: 5′-TGG​TAC​CAT​TGA​GTT​GCT​GCT​ATT-3′ and 5′-AGC​TCA​TGT​GTT​GCC​CTT​TAT​ACA-3′, HOXA1: 5′-CAG​CGC​AGA​CTT​TTG​ACT​GGA​TG-3′ and 5′-TCC​TTC​TCC​AGT​TCC​GTG​AGC​T-3′, ISCU: 5′-GGG​TCC​CTT​GAC​AAG​ACA​TCT-3′ and 5′-CCT​TTC​ACC​CAT​TCA​GTG​GCT​A-3′, MNT: 5′-ACG​TAC​TGG​AGA​TTG​ACC​GCG​T-3′ and 5′-CCT​CGT​CTA​TGT​TGT​CCT​CAC​C-3′, and BNIP3L: 5′-AAT​GTC​GTC​CCA​CCT​AGT​CG -3′ and 5′-CCC​CCA​TTT​TTC​CCA​TTG​CC-3’.

### Cellular phenotype analyses

All experiments had at least three biological replicates. The MTT (thiazolyl blue tetrazolium bromide, catalog number A600799, Sangon) assay to measure cell proliferation and the scratch assay to examine cell migration had been described (Zhang et al., 2013). For MTT assay 3,000 cells were seeded per well in a 96-well plate, and cell numbers measured once every 24 h. For time points longer than 3 days, cells were split 1:8 on day 3 for continuous studies. For scratch assay, cells were grown in a 24-well plate to over 80% confluency, then a pipet tip was used to make a straight line scratch across the cell monolayer surface. After floating cells were removed, the remaining cells were cultured in serum-free media for the next 24 h. At 0, 12, and 24 h the scratch or the gap it introduced was monitored under a light microscope, with cells entering the gap region considered migration.

The Cell Cycle and Apoptosis Analysis Kit (YEASEN, Shanghai, China) was used to study the cell cycle profiles. Approximately 2 × 10^5^ cells were washed and collected by centrifugation at 1,000 g for 5 min. The cells were fixed in cold 70% ethanol, washed, and stained with a propidium iodide (PI) solution containing RNase A. The stained cells were filtered through a 400-mesh sieve and analyzed using a BD Accuri C6 flow cytometer and the FlowJo v10.6 software (Becton Dickinson, Franklin Lakes, NJ, United States). Cells at different phases of the cell cycle were determined automatically using the default settings. The Annexin V-Alexa Fluor 647/PI Apoptosis Detection Kit (YEASEN) was used to study apoptosis. Harvested cells were stained with Annexin V-Alexa Fluor 647 and PI and analyzed using a BD Accuri C6 flow cytometer and the FlowJo v10.6 software as described above.

Reactive oxygen species (ROS) levels were measured using the Meilun Reactive Oxygen Species Assay Kit (Meilunbio, Dalian, Liaoning, China). The kit uses 2,7-Dichlorodi-hydrofluorescein diacetate, a dye that fluoresces when oxidized by ROS. Approximately 1 × 10^5^ cells were stained with the dye for flow cytometry studies, and cells were excited at 488 nm and detected at 525 nm. Fluorescence signals were quantified using the FlowJo v10.6 software. Alternatively, cells grown in a 6-well plate were stained with the dye and monitored under a microscope. The mitochondrial membrane potential was measured using the JC-1 Mitochondrial Membrane Potential Assay from Sciben (Nanjing, China), per manufacture’s instructions. JC-1 is a dye that accumulates in the mitochondria. When the membrane potential is low, JC-1 exists as a monomer and produces green fluorescence. When the membrane potential is high, JC-1 aggregates and yields red fluorescence. Hence the ratio of red vs. green fluorescence can be an index of mitochondrial membrane potential changes. Approximately 1-6 × 10^5^ cells were stained with JC-1 solution, washed, and then analyzed using a BD Accuri C6 flow cytometer. Cells were excited at 490 nm and 525 nm and detected at 530 and 590 nm, respectively. The FlowJo v10.6 software was used to quantify red and green fluorescence signals automatically.

### High-throughput RNA sequencing (RNA-seq) and data analyses

For RNA-seq (Azenta Life Sciences, Burlington, MA, Unites States), a cDNA library was prepared from 1 µg of 293T total RNA. Briefly, poly(A) mRNA was isolated by oligo (dT) beads and fragmented with divalent cations at high temperature, before cDNA synthesis. cDNA was tailed with dA, followed by T-A ligation to add adapters, which was then amplified by PCR and loaded onto an Illumina HiSeq/Illumina Novaseq/MGI2000 instrument for sequencing with a 2 × 150 paired-end configuration, according to the manufacturer’s instructions (Illumina, San Diego, CA, United States). Raw sequence data were processed by Cutadapt (V1.9.1), and the clean data aligned to the human reference genome GRCh38 with Hisat2 (v2.0.1). Gene expression levels were obtained using HTSeq (v0.6.1). RNA-seq data have been deposited in the Gene Expression Omnibus under the accession number GSE243063. Student’s t-test was performed to identify differentially expressed genes from three biological replicates (*p* < 0.05), and DAVID (https://david.ncifcrf.gov) (Sherman et al., 2022) used to analyze differences in GO (Gene Ontology) TERM-BP (biological processes)-Direct (GO_BP_Direct for short) and KEGG (Kyoto Encyclopedia of Genes and Genomes) pathways.

### Protein analyses

Cell lysis, protein extraction, and Western blotting were performed as described (Zhang et al., 2013). The primary FLAG antibody was from Sigma (dilution 1:5,000, catalog number F3165, St Louis, MO, United States), GAPDH antibody from Solarbio (dilution 1:2000, catalog number K200057M, Beijing, China), and BNIP3L antibody from Beyotime (dilution 1:2000, catalog number AF63333). For reporter assays, 293T cells were transfected with approximately 10 ng of a firefly luc reporter plasmid, 5 ng of pRL-CMV, and 400 ng of a control or miR-210 mimic RNA. Two days later, the cells were lyzed, and firefly and Renilla luc activities measured using the Dual-Luciferase Assay System (Promega).

### Statistical analysis

GraphPad Prism 7.0 (GraphPad Software, San Diego, CA, United States) was used to perform the two-sided, two-sample unequal variance Student’s t-test and Dunnett’s test, with *p*-value <0.05 considered statistically significant.

## Results

### Generation of miR-210 KO 293T cells using the CRISPR/Cas9 technology

**FIGURE 1:**
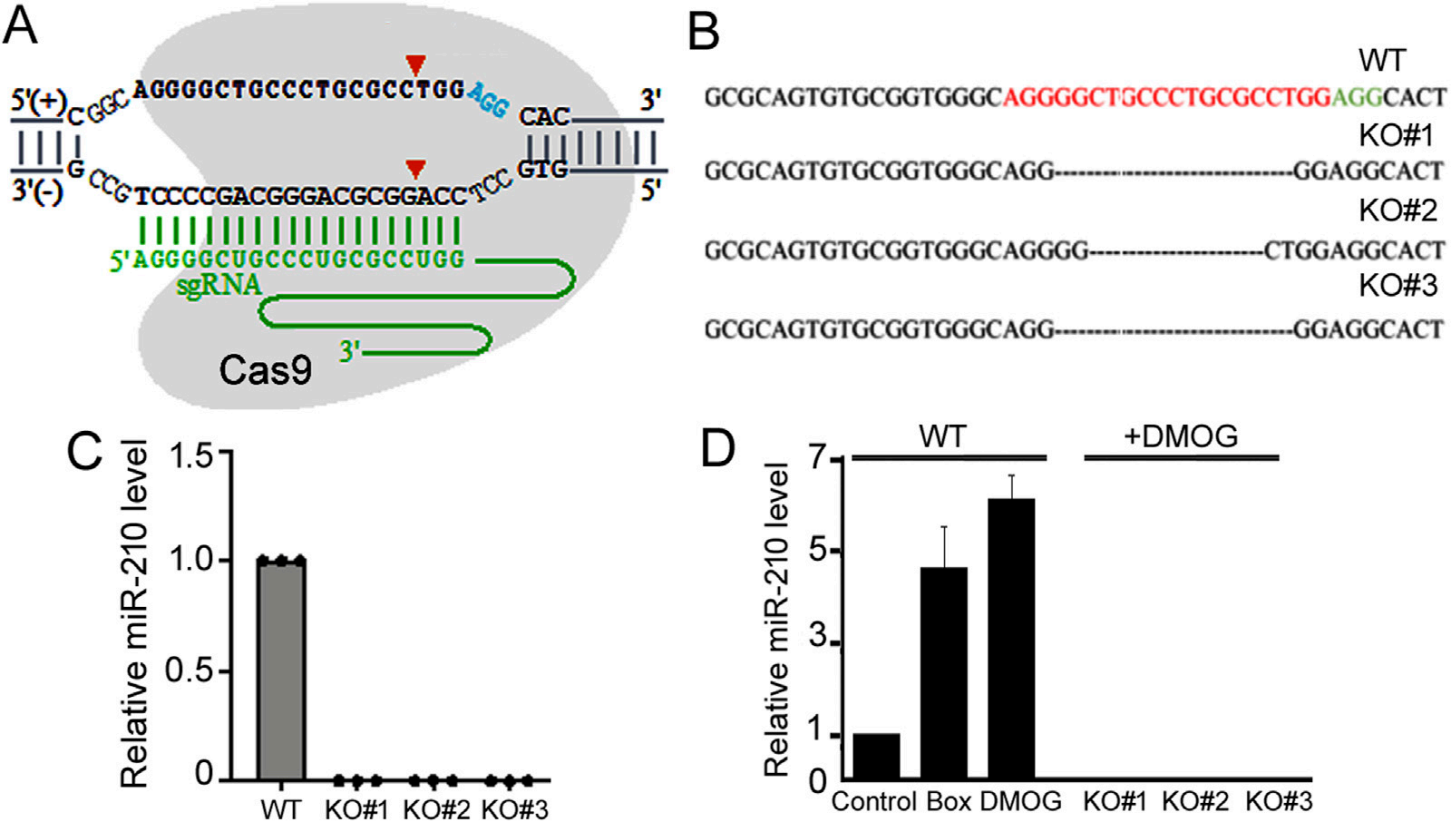
Establishment of 293T *miR-210* KO cell lines. **(A)** Schematic representation of the sgRNA targeting the *miR-210* gene locus. The protospacer adjacent motif sequence is in blue, sgRNA sequence in green, and Cas9 cleavage sites shown by arrowheads. The sgRNA does not need to target the entire mature miR-210 sequence, as the loss of surrounding DNA/RNA would also disrupt miRNA maturation. **(B)** Sanger sequencing results of the *miR-210* locus in the WT and KO cells. The protospacer adjacent motif sequence is shown in green, and the *miR-210* gene locus sequence corresponding to the sgRNA in red. **(C)** qPCR analysis of relative miR-210 expression in WT and KO cells under normal growth conditions. The expression in WT cells was set at 1.0. **(D)** qPCR analysis of relative miR-210 expression in the WT and KO cells under normoxic and hypoxic conditions. The expression in the WT cells under normoxic condition was set at 1.0. Averages and standard deviations are shown. Box: cells were placed in a hypoxia chamber.

To target and disrupt the human *miR-210* gene, we designed an sgRNA as depicted in Figure 1A and constructed the vector LentiCRISPR-miR-210 (**MATERIALS AND METHODS**). We then transfected 293T cells with LentiCRISPR-miR-210, which co-expressed the sgRNA and Cas9 protein. Controls (WT) were transfected with the empty vector LentiCRISPR DNA. Clonal cells were selected, and one WT clone and three KO clones (#1, 2, and 3) were acquired and amplified. The loss of *miR-210* was identified and confirmed by PCR, DNA sequencing, and qPCR. At the *miR-210* gene locus KO#1 and 2 had slight differences in the DNA deletion, while KO#3 was the same as KO#1 (Figure 1B). And qPCR results verified all three had no discernible miR-210 RNA expression (Figure 1C).

The CRISPR/Cas9 system can induce off-target mutations. To further characterize our KO cells, we obtained the top 11 potential off-target sites in the human genome based on the sgRNA sequence (Supplementary Table S1). Genomic DNA for these sites was amplified and sequenced. The results revealed that none of the KO cell lines contained mutations in those loci (Supplementary Table S2).

Considering the relationship between miR-210 and hypoxia response, we next measured miR-210 expression under hypoxia. Hypoxia response can be induced by placing cells in a low oxygen chamber or mimicked by treating cells with DMOG, a classic drug that stabilizes HIF-1ɑ (Jaakkola et al., 2001). Both methods significantly elevated miR-210 levels in the WT 293T cells (Figure 1D). For simplicity we would use DMOG to mimic hypoxia thereafter. In the KO cells, as expected, miR-210 expression is undetectable under both the normoxic and hypoxic conditions (Figures 1C,D). Since all three KO cell lines (#1, 2, and 3) exhibited consistent results (Figures 1B–D), we would use two of them (#1 and 2) for subsequent experiments.

### Characterizing the phenotypes of miR-210 KO cells

**FIGURE 2:**
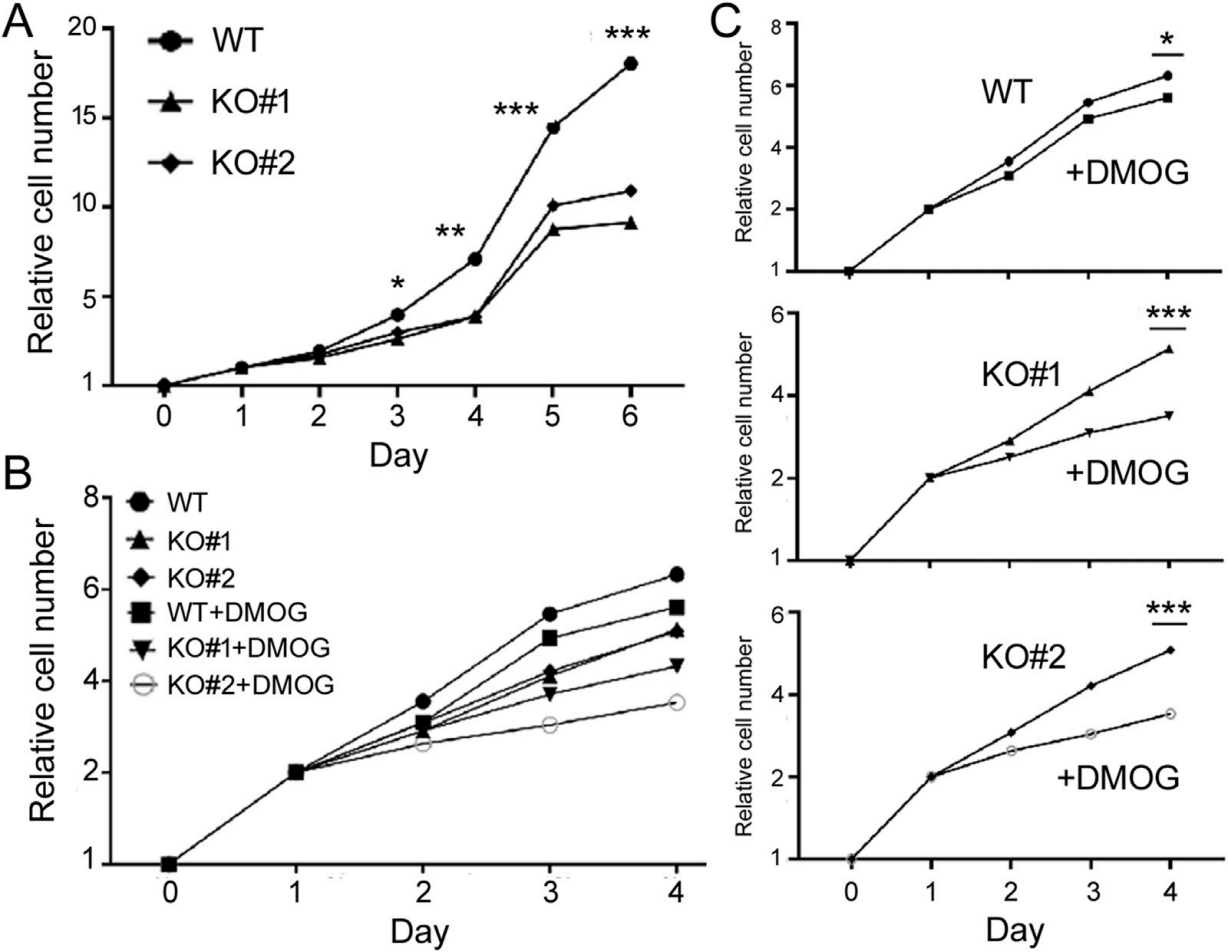
Growth of the WT and miR-210 KO cells measured using the MTT method. **(A)** Growth under the normoxic condition. The *y*-axis shows the relative cell number (1 on day 0), and the *x*-axis times in days. Symbols represent average cell numbers, with standard deviations too small to indicate on the graphs. Comparing WT and KO cell numbers, *: *p* < 0.05; **: *p* < 0.01; ***: *p* < 0.001. **(B)** Growth under the normoxic or DMOG treatment conditions. The *y*-axis shows the relative cell number (1 on day 0), and the *x*-axis times in days. **(C)** Separate comparisons of the WT and KO cells under normoxic and hypoxic conditions based on **(B)**. Comparing DMSO and DMOG treatments at the last time point, *: *p* < 0.05; ***: *p* < 0.001.

**FIGURE 3:**
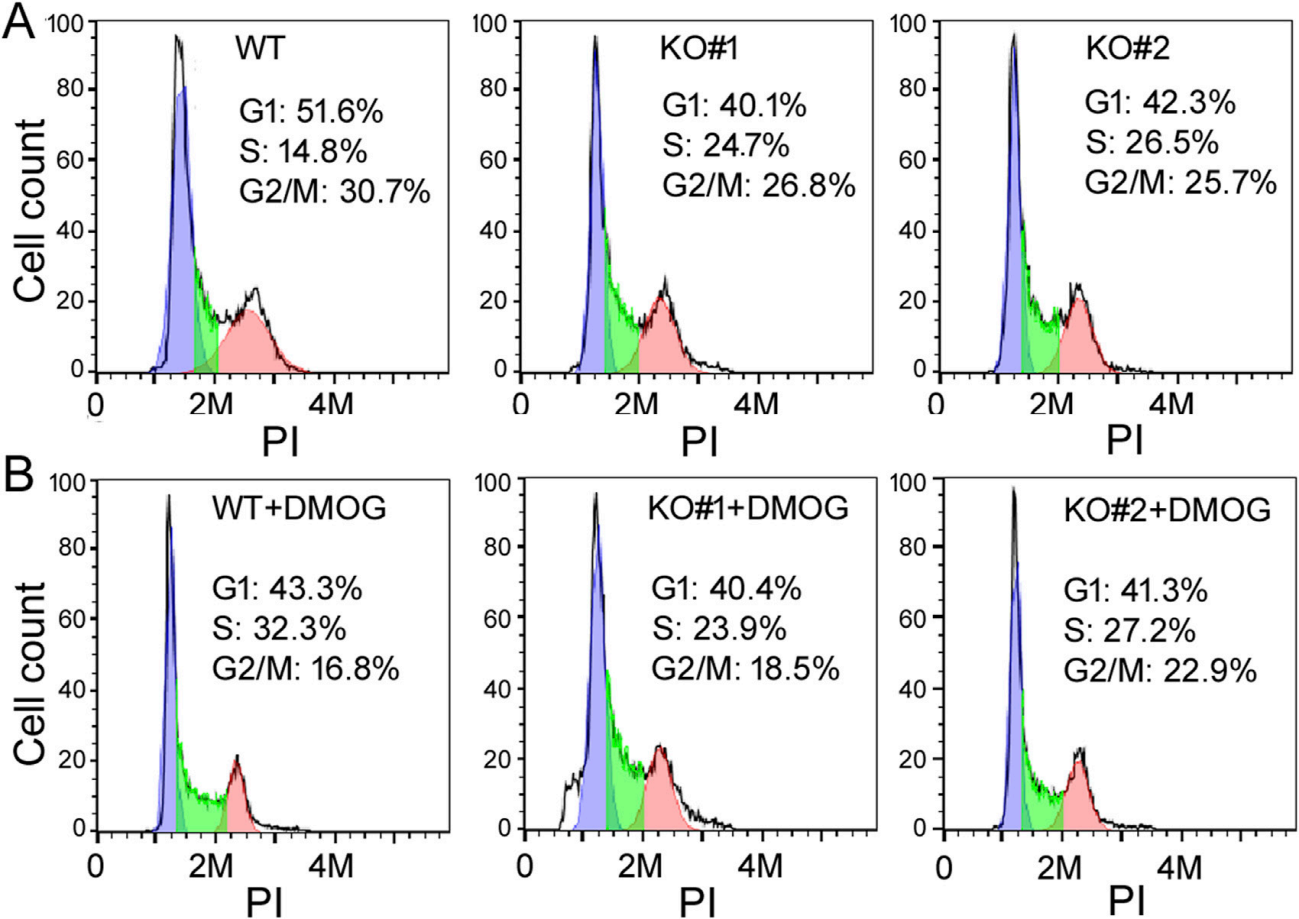
The cell cycle profiles of WT and KO cells under control **(A)** and DMOG treatments **(B)**. The y-axis is the cell count, the *x*-axis PI staining that measured DNA content. Representatives of at least three experiments are shown here. The percentages of cells in the G1, S, and G2/M phases were determined automatically by the default settings of the flow cytometry and software and listed inside the graphs.

To understand the functions of miR-210, we then investigated the impact of *miR-210* KO on 293T cell behaviors, including growth, migration, apoptosis, and metabolism. We first used MTT assays to measure cell number increase, or cell growth. Under normal oxygen conditions, KO cells exhibited slower growth than the WT cells (Figure 2A). DMOG-induced hypoxia responses hindered cell proliferation in both the WT and KO cells (Figures 2B, C). We also examined their cell cycle profiles by flow cytometry. Compared to the WT cells, more KO cells were at the S phase but fewer at the G2/M phase (30.7% G2/M for the WT, 26.8% and 25.7% for KO, Figure 3A), while DMOG appreciably reduced WT cells in the G2/M phase (Figure 3B). These results were consistent with the MTT data suggesting that KO and DMOG both reduced cell numbers.

**FIGURE 4:**
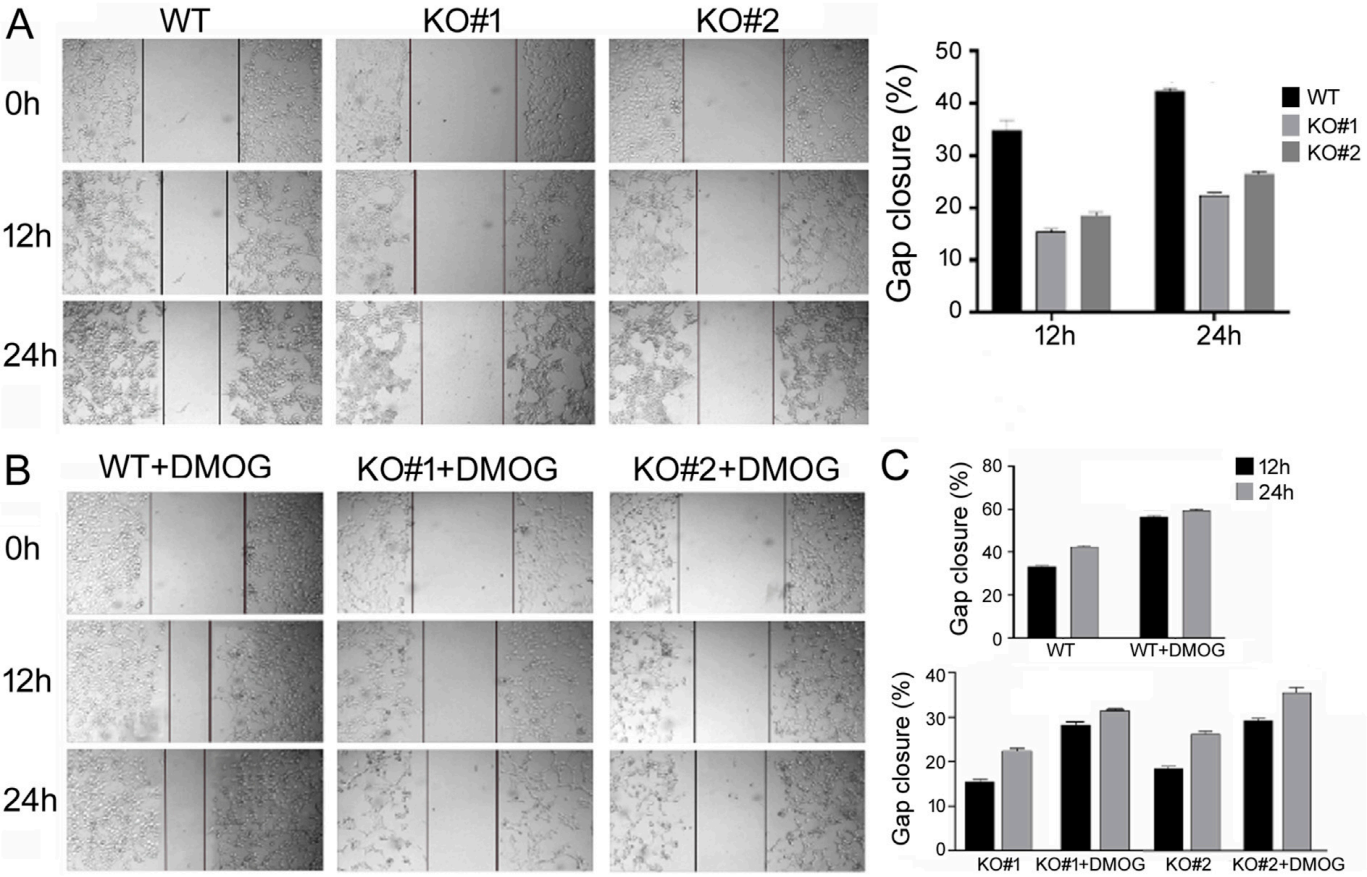
Scratch assays to measure migration of the WT and KO cells. **(A)** Migration under normoxic condition. Representative cell images are shown, with lines marking the migrating, leading edges of cells. The right panel shows the quantification on gap closure or migration at the 12 h and 24 h time points. Gap closure was defined as one minus the ratio of the gap width at 12 or 24 h vs. that at 0 h when the scratch was made and presented in percentage. Averages and standard deviations are shown. **(B)** Representative cell images under DMOG treatment. **(C)** Quantification and comparison of gap closure of the WT and KO cells under normoxic and DMOG conditions. Averages and standard deviations are shown.

Next we used scratch assays to study cell migration, which is important for biological processes such as development, wound healing, angiogenesis, and metastasis. Under serum-free growth conditions, KO cells migrated slower into the gap region than the WT cells with both DMSO treatment (Figure 4A) and DMOG treatment (Figure 4B). Comparisons of the quantifications are presented in Figure 4C, also showing that DMOG/hypoxia stimulated migration in both the WT and KO cells.

**FIGURE 5:**
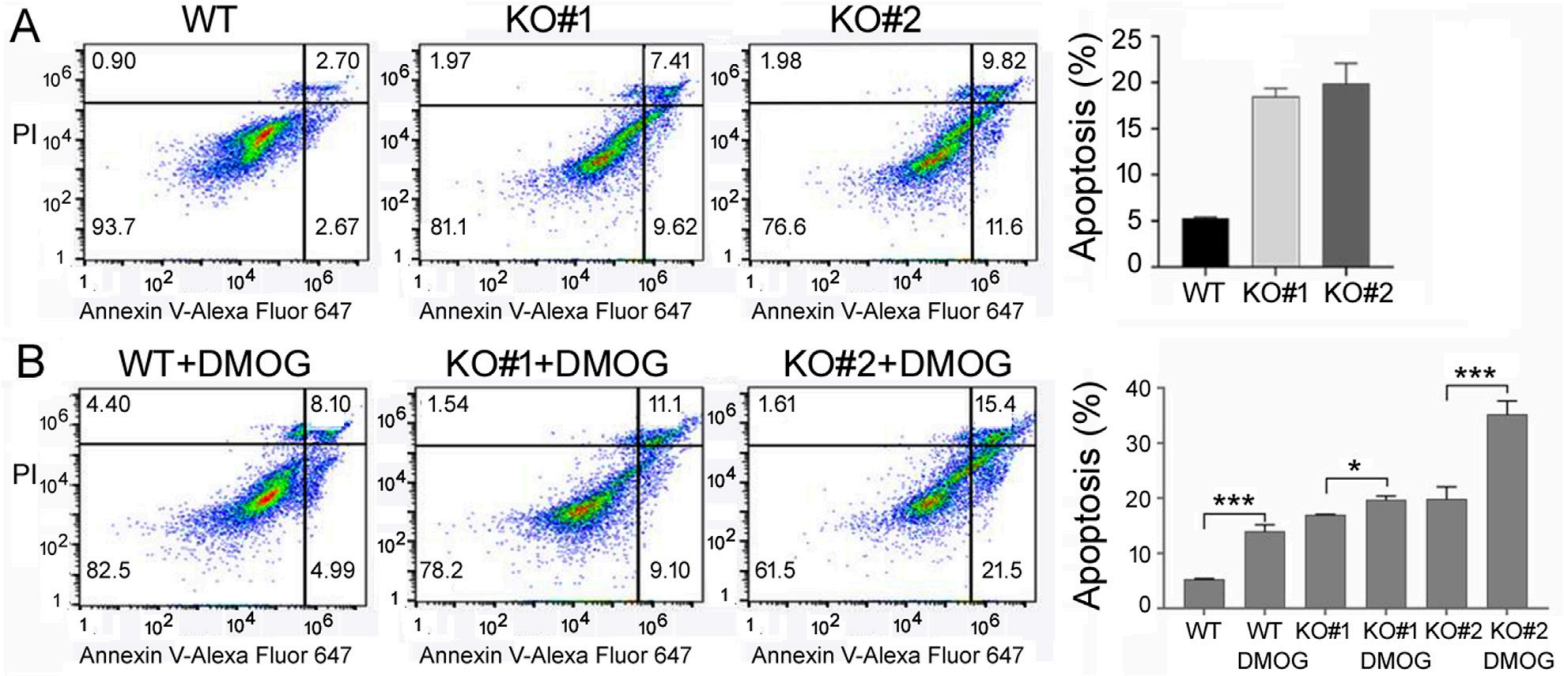
Apoptosis of the WT and KO cells under normoxic **(A)** and DMOG **(B)** conditions. PI staining (the *y*-axis) detected DNA content, and Annexin V staining (the *x*-axis) examined apoptosis, which was represented by the lower right quadrant, corresponding to early apoptotic cells, and the upper right quadrant, corresponding to late apoptotic cells. Percentages of cells in each quadrant were determined automatically by the default settings of the flow cytometry and software and listed inside the graphs. Right panels show the average and standard deviations of the percentages of total apoptotic cells. *: *p* < 0.05; ***: *p* < 0.001.

**FIGURE 6:**
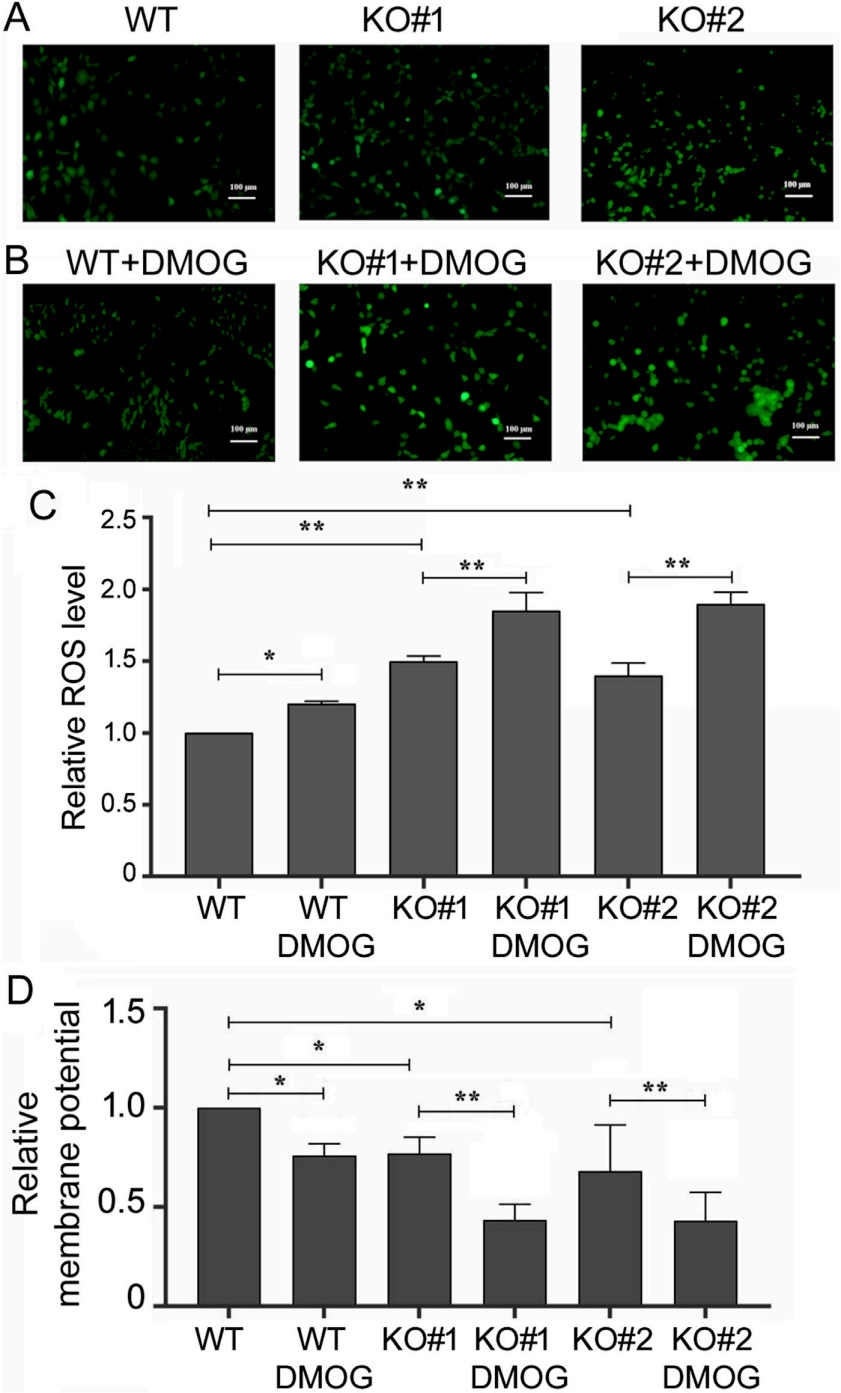
ROS levels and mitochondrial membrane potentials in the WT and KO cells. **(A)** Cells under normoxic condition after staining with 2,7-Dichlorodi-hydrofluorescein diacetate, which measured ROS levels and emitted green fluorescence. Ruler size is included in the images. **(B)** Green fluorescence of the cells measuring ROS levels, as in **(A)**, under DMOG treatment. **(C)** Quantification of relative ROS level (the *y*-axis) as defined by the intensity of the green fluorescence, with that of the WT cells under normoxic condition set at 1. Averages and standard deviations are shown. *: *p* < 0.05; **: *p* < 0.01. **(D)** Quantification of relative mitochondrial membrane potential (the *y*-axis), as defined by the intensity of the red fluorescence divided by that of the green fluorescence, after staining with JC-1 and measured by cell flow cytometry automatically. WT cells under normoxic conditions were set at 1. Averages and standard deviations are shown. *: *p* < 0.05; **: *p* < 0.01.

We then examined apoptosis in 293T cells by Annexin V staining and flow cytometry. High Annexin V signals identified apoptotic cells, and we found that *miR-210* KO cells had a higher apoptotic ratio compared to the WT cells (Figure 5A), and DMOG increased apoptosis in both the WT and KO cells (Figure 5B). As mitochondrial functions and metabolism are closely linked to oxygen and hypoxia as well as to apoptosis, we also measured the intracellular ROS levels and relative mitochondrial membrane potential. We found that KO cells had higher ROS content (Figure 6A), whereas DMOG treatment enhanced ROS in both the WT and KO cells (Figures 6B, C). Compared to WT cells, KO cells had lower mitochondrial membrane potential, and DMOG led to a further drop in the membrane potential in all cells (Figure 6D).

### Transcriptome analyses of miR-210 KO 293T cells

As shown above, *miR-210* KO perturbed a wide range of phenotypes in 293T cells. To begin to unravel the underlying mechanisms, we performed poly(A) RNA-seq on *miR-210* WT and KO cells under control and DMOG treatment conditions. Differentially expressed genes were obtained from three independent experiments and then subject to GO and KEGG pathway analyses. We first compared the KO vs. WT cells under normal growth conditions (Table 1). The top GO term differences prominently featured transcription and DNA replication, and the top KEGG pathway differences included metabolic pathways and DNA replication. The DNA replication and metabolic pathways findings were in line with the cell growth and cell cycle phenotypes we observed in Figures 2, 3 as well as the putative link between miR-210/hypoxia response and metabolism.

**TABLE 1:** GO and KEGG pathway analyses of the differentially expressed genes in *miR-210* KO cells vs. WT cells under normal growth conditions. Top 10 results are listed along with the P values.

GO and KEGG terms	P value
GO_BP_Direct	
positive regulation of transcription, DNA-templated	2.5E-10
positive regulation of transcription from RNA polymerase II promoter	3.5E-8
negative regulation of transcription from RNA polymerase II promoter	9.4E-6
chromatin organization	1.1E-5
chromatin remodeling	1.6E-5
negative regulation of transcription, DNA-templated	2.2E-5
protein phosphorylation	2.7E-5
DNA replication	2.8E-5
anterior/posterior pattern specification	3.1E-5
actin cytoskeleton organization	3.3E-5
KEGG	
Metabolic pathways	3.7E-4
N-Glycan biosynthesis	3.7E-4
Phosphatidylinositol signaling system	3.0E-3
Inositol phosphate metabolism	3.6E-3
Amoebiasis	6.1E-3
Lysosome	9.1E-3
melanogenesis	1.1E-2
DNA replication	1.2E-2
Pathways in cancer	1.2E-2
Aldosterone synthesis and secretion	1.4E-2

Secondly, we compared normal vs. DMOG, i.e., hypoxia-mimicking conditions. Data from the WT cells yielded GO terms implicating the development of multiple organs and wound healing, all known associations with hypoxia responses (Table 2), but few KEGG terms, likely because there were only three WT replicate samples. Thus we also looked at the more numerous KO samples, since hypoxia response was maintained in KO cells as well (see below and **DISCUSSION**). This analysis demonstrated that hypoxia led to changes in GO terms such as translation, metabolism, and DNA replication initiation (organ development also present but outside of the top 10), and KEGG including numerous metabolic pathways, the HIF-1 signaling pathway, and the cell cycle (Table 2).

**TABLE 2:** GO and KEGG pathway analyses of the differentially expressed genes induced by DMOG/hypoxia. Top 10 results are listed along with the P values.

GO and KEGG terms	P value
GO_BP_Direct, WT cells	
animal organ development	1.3E-4
endochondral bone growth	3.5E-3
cartilage development involved in endochondral bone morphogenesis	3.5E-3
regulation of blood pressure	1.7E-2
heart morphogenesis	1.7E-2
positive regulation of transcription from RNA polymerase II promoter	2.3E-2
chromatin remodeling	2.7E-2
negative regulation of cell migration	3.1E-2
positive regulation of protein complex assembly	3.1E-2
wound healing	3.2E-2
GO_BP_Direct, KO cells	
mitochondrial translation	1.2E-9
sphingolipid biosynthetic process	7.9E-4
lysosome localization	1.3E-3
cellular response to BMP stimulus	1.3E-3
carbohydrate derivative biosynthetic process	1.3E-3
proximal/distal pattern formation	1.6E-3
natural killer cell activation	1.6E-3
DNA replication initiation	2.6E-3
translation	6.4E-3
canonical glycolysis	6.6E-3
KEGG, KO cells	
Metabolic pathways	2.8E-5
Glycolysis / Gluconeogenesis	1.7E-3
Carbon metabolism	1.8E-2
p53 signaling pathway	2.7E-2
Glycosaminoglycan biosynthesis - heparan sulfate / heparin	3.5E-2
RNA degradation	4.1E-2
HIF-1 signaling pathway	4.3E-2
Fatty acid elongation	5.4E-2
Glycosaminoglycan degradation	5.5E-2
Cell cycle	6.2E-2

Lastly, we examined changes in hypoxia responses, defined as the expression of a gene under DMOG treatment divided by that under DMSO treatment, in KO cells vs. WT cells. GO term differences included mitochondrial respiratory chain complex IV assembly, morphogenesis, response to wounding, and kidney development, and KEGG included a large number of metabolic pathways and apoptosis (Table 3). These processes are likely the ones impacted by miR-210 during hypoxia.

**TABLE 3:** GO and KEGG pathway analyses of the DMOG-induced, differentially response genes in *miR-210* KO cells vs. WT cells. Top 10 results are listed along with the P values.

GO and KEGG terms	P value
GO_BP_Direct	
mitochondrial respiratory chain complex IV assembly	5.6E-4
regulation of transcription from RNA polymerase II promoter	1.6E-3
regulation of blood pressure	3.0E-3
embryonic limb morphogenesis	3.3E-3
pharyngeal arch artery morphogenesis	3.8E-3
response to wounding	4.2E-3
kidney development	4.2E-3
response to xenobiotic stimulus	4.3E-3
positive regulation of transcription from RNA polymerase II promoter	4.5E-3
branching morphogenesis of an epithelial tube	4.6E-3
KEGG	
Metabolic pathways	3.3E-3
TNF signaling pathway	3.7E-3
Apoptosis - multiple species	7.3E-3
Biosynthesis of cofactors	1.6E-2
Pathogenic Escherichia coli infection	1.7E-2
Pathways in cancer	1.8E-2
Fatty acid metabolism	2.2E-2
Purine metabolism	2.4E-2
AGE-RAGE signaling pathway in diabetic complications	3.0E-2
Fatty acid biosynthesis	3.8E-2

Top 10 results are listed along with the P values.

### miR-210 KO cells have increased BNIP3L expression

**FIGURE 7:**
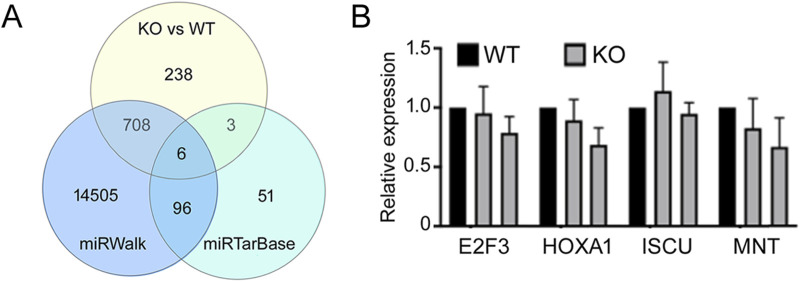
Analyses of miR-210 target genes. **(A)** Venn diagram of the intersections among genes that had increased expression upon miR-210 KO in 293T cells, miR-210 target genes predicted by miRWalk, and experimentally validated genes according to miRTarBase. **(B)** qPCR analysis of the indicated mRNAs. Expression in the WT cells was set at 1. Averages and standard deviations are shown. Differences between the WT and KO expression were not significant (*p* > 0.05).

Animal miRNAs bind to complementary sequences in target mRNAs to repress the latter’s expression (Bartel, 2018), so *miR-210* KO was expected to result in increased expression of miR-210 target genes and mRNAs. To identify these target genes we focused on mRNAs that were expressed higher in the KO cells than WT cells. There were 955 such genes (*p* < 0.05; Supplementary Table S3). We then compared them with the miRNA target prediction site miRWalk (Sticht et al., 2018) and the experimentally validated miRNA target site miRTarBase (Huang et al., 2022). Of the 955 genes, 714 were predicted by miRWalk (Figure 7A; Supplementary Table S3). GO terms of these genes included cell migration, angiogenesis, negative regulation of cell proliferation, and response to wounding, and many KEGG pathways were related to adhesion, human cancers and diabetes (Table 4), again consistent with KO cell phenotypes (Figures 2–4) and the presumed functions of miR-210 in disease and metastasis. On the other hand, there were 156 validated mouse and human miR-210 targets in miRTarBase, but only 9 were upregulated in KO cells (Figure 7A; Supplementary Table S3). Since many reported miR-210 target genes, such as *EFNA3, HOXA1, PTP1B, PLK1, CDC25B, CCNF, BUB1B, MNT, FAM83D, E2F3, CASP8AP2, AIFM3, BNIP3, SIN3A,* and *ISCU* have important functions (**INTRODUCTION**), we checked their changes individually and found that only *PTP1B, CDC25B,* and *FAM83D* significantly increased mRNAs upon *miR-210* KO in 293T cells (Supplementary Table S3). For the other genes we selected *E2F3, HOXA1, ISCU,* and *MNT* for additional qPCR verification and confirmed they were not upregulated (Figure 7B). Thus, *miR-210* KO did not enhance mRNA levels of the vast majority of reported miR-210 target genes, at least in 293T cells.

**TABLE 4:** GO and KEGG pathway analyses of the putative miR-210 target genes upregulated in *miR-210* KO cells. Top 10 results are listed along with the P values.

GO and KEGG terms	P value
GO_BP_Direct	
cell migration	2.1E-7
angiogenesis	6.1E-6
protein phosphorylation	1.6E-4
negative regulation of cell proliferation	4.2E-4
cell differentiation	6.3E-4
regulation of transcription from RNA polymerase II promoter	1.0E-3
epithelial to mesenchymal transition	1.5E-3
somatic stem cell population maintenance	2.1E-3
canonical Wnt signaling pathway	2.3E-3
response to wounding	2.6E-3
KEGG	
ECM-receptor interaction	1.5E-4
Amoebiasis	5.5E-4
Small cell lung cancer	8.1E-4
AGE-RAGE signaling pathway in diabetic complication	1.6E-3
Pathways in cancer	2.5E-3
Fluid shear stress and atherosclerosis	2.8E-3
Toxoplasmosis	4.0E-3
Proteoglycans in cancer	6.0E-3
Maturity onset diabetes of the young	1.2E-2
Focal adhesion	1.7E-2

**FIGURE 8:**
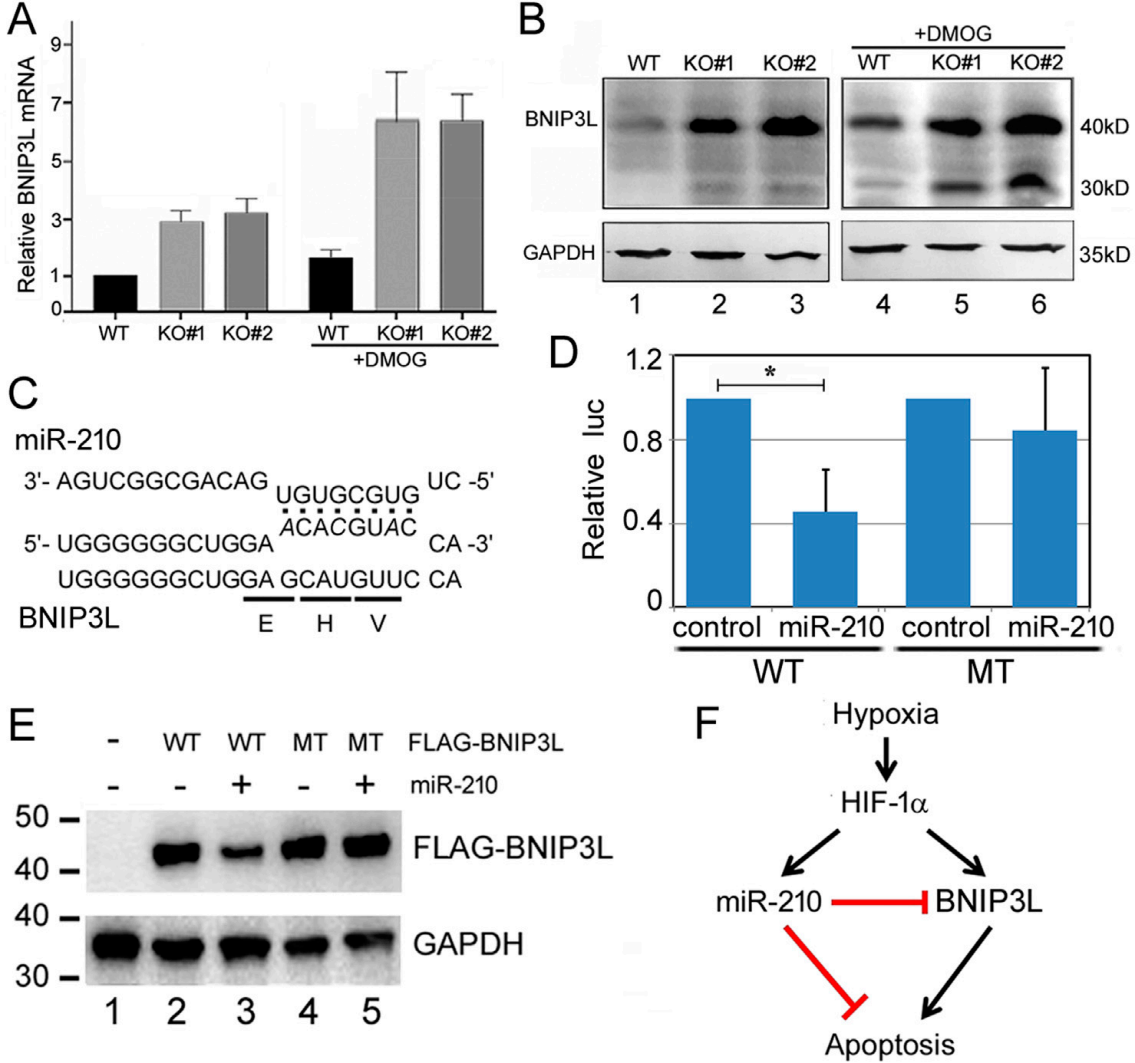
BNIP3L is a miR-210 target. **(A)** qPCR quantification of BNIP3L mRNA levels. Expression in the WT cells at normoxia was set to 1. Averages and standard deviations are shown. **(B)** BNIP3LWestern blot analysis. GAPDH was the loading control. Expected molecular weights of the proteins are indicated on the right. Lanes 1-3: control condition; lanes 4-6: DMOG treatment condition. **(C)** Predicted miR-210 binding site in BNIP3L mRNA (NM_004331, nucleotides 238-260). miR-210 sequence is indicated on top. The encoded amino acids and mutant BNIP3L mRNA sequences are indicated at the bottom. **(D)** Effects of miR-210 on the expression of WT and MT BNIP3L luc reporters in 293T cells. *Y*-axis is the relative ratio of the firefly luc activity to the Renilla luc activity, with the control RNA transfection conditions set at 1. The averages and standard deviations are indicated. *: *p* < 0.05. **(E)** Western blot analysis of FLAG-BNIP3L (WT and MT) expression with or without miR-210 in 293T cells. Transfection conditions are indicated on top. FLAG-BNIP3L was detected using a FLAG antibody, and GAPDH served as the loading control. This experiment was preformed independently four times, with representative results shown here. **(F)** A model depicting the interplay among hypoxia, HIF-1α, miR-210, BNIP3L, and apoptosis. HIF-1α and miR-210 have other targets that are not shown here.

The major phenotypes of *miR-210* KO cells were reduced cell proliferation and enhanced apoptosis (Figures 2, 3, 5), with ROS and mitochondrial membrane potential changes potentially related to or associated with such cellular phenotypes as well (Figure 6). Gene expression, GO, and KEGG pathway analyses supported these observations, although the mechanisms required experimental validation. As the first step we focused on understanding how miR-210 suppressed apoptosis in 293T cells. We noticed that rat *BNIP3*, a pro-apoptotic member of the *Bcl-2* family (Field and Gordon, 2022), is induced by hypoxia and has been reported to be a miR-210 target (Wang et al., 2013). BNIP3 mRNA was increased in our KO cells, although the change was not statistically significant (*p* > 0.05), but the mRNA increase of *BNIP3L* or *Nix*, a *BNIP3* relative (Field and Gordon, 2022), was significant, and *BNIP3L* is predicted by miRWalk as a miR-210 target (Supplementary Table S3). So we performed qPCR experiments, which confirmed that BNIP3L mRNA was indeed elevated in KO cells (Figure 8A). DMOG treatment increased BNIP3L mRNA levels in both the WT and KO cells (Figure 8A), consistent with the report of BNIP3L responding to hypoxia (Bruick, 2000).

We next examined BNIP3L protein expression by Western blotting (Figure 8B). BNIP3L ran as a 40kD protein on the blot, with a 30kD band probably representing either an isoform or BNIP3 (Field and Gordon, 2022). KO#1 and KO#2 cells expressed a higher level of BNIP3L protein, compared to the WT cells (Figure 8B, lanes 1-3). Figure 8B further shows that in WT cells, DMOG increased BNIP3L protein levels (compare lanes 1 and 4), and under these same conditions and compared to WT cells, KO cells still had higher BNIP3L protein expression (compare lanes 5 and 6 with 2 and 3). Unusually, the predicted miR-210 binding site is in the coding region of BNIP3L mRNA (Figure 8C). To test if miR-210 can inhibit BNIP3L expression, we first employed a conventional reporter assay. As shown in Figure 8D, the expression of a luc reporter containing WT BNIP3L sequence at its 3′ untranslated region was reduced approximately 60% by miR-210 in co-transfected 293T cells, while a mutant (MT) BNIP3L reporter was not significantly repressed by miR-210. Next, to further confirm that miR-210 was able to directly suppress BNIP3L expression, we constructed a FLAG-tagged BNIP3L and its mutant version with three nucleotide mutations that code for the same amino acids but should weaken the binding to miR-210 (Figure 8C). Cotransfecting miR-210 with the WT plasmid led to approximately 50% reduction in FLAG-BNIP3L protein expression (Figure 8E, compare lanes 2 and 3). miR-210 had no effect on the MT protein expression (Figure 8E, compare lanes 4 and 5). A model was proposed and presented in Figure 8F to illustrate how miR-210 through BNIP3L might counteract the effects of hypoxia and HIF-1α on apoptosis.

## Discussion

In this study we constructed *miR-210* gene KO in 293T cells and used these cells to investigate the functions and mechanisms of human miR-210. We showed that *miR-210* KO reduced cell growth, migration, and mitochondrial membrane potential, while enhancing apoptosis and cellular ROS levels. Transcriptome analyses identified changes in biological processes and pathways that could explain the aforementioned phenotypes as well as putative miR-210 functions in organism development, metabolism, and human diseases. Lastly, we identified BNIP3L as a potential mediator of miR-210 function in apoptosis.

A number of miR-210 target genes have been reported (Huang et al., 2022). On the other hand, *miR-210* KO mice have only mild phenotypes, which fails to paint a clear picture about the relationship between miR-210 and those target genes or hypoxia (Wang H. et al., 2014; Mok et al., 2013; White et al., 2015; Krawczynski et al., 2016; Bian et al., 2021; Watts et al., 2021). Perhaps this is consistent with the “fine-tuning” mechanisms of action often ascribed to animal miRNAs (Bartel, 2018; Ivan and Huang, 2014). Furthermore, many reports yielded conflicting results with regard to cellular phenotypes and miR-210s expression and roles in cancers, and cross validation of miR-210 targets or systematic studies of the human miR-210 functions are lacking. So it is unclear whether miR-210 has biological functions only under hypoxia or in normoxia as well, and what those functions may be, especially in humans. Our work showed that *miR-210* KO in 293T cells resulted in a series of phenotypes in cell proliferation, migration, apoptosis, and changes in the mitochondrial membrane potential and ROS levels (Figures 2–6), indicating that human miR-210 has a constitutive role under both normoxic and hypoxic conditions. The phenotypes of cell growth, migration, and apoptosis we observed could be interconnected, as the slower cell number increase in KO cells (Figure 2) might be due to a combination of reduced cell division and increased cell death, which could similarly impact KO cell migration (Figure 4). Thus, changes in 293T behaviors due to miR-210 loss, such as the cell cycle progression and apoptosis, and the underlying mechanisms deserve more in-depth studies in the future. On the other hand, the loss of miR-210 did not abrogate hypoxia responses, consistent with the fact that miR-210 is merely one of the many HIF targets. We conclude that at least in 293T cells miR-210 contributes positively to cell growth and survival. miR-210 had been reported to either enhance (e.g., (Rothé et al., 2011)) or inhibit (e.g., (Tsuchiya et al., 2011)) cell proliferation, enhance (e.g., (Tsuchiya et al., 2011)) or inhibit apoptosis (e.g., (Fasanaro et al., 2008)), and enhance (e.g., (Chen et al., 2010)) or inhibit ROS formation (e.g., (Li et al., 2017)). These discrepancies may be due to the different cell types and experimental systems employed. Previous studies either overexpressed miR-210 or inhibited miR-210 with an antagomir, which could affect non-physiological targets. Our study differed from earlier work by using human *miR-210* KO clones but otherwise examined cells under common culture conditions.

By global transcriptomics analyses we found that the KO cells differed from WT cells in biological processes and pathways such as DNA replication, metabolic pathways, apoptosis, response to wounding (Tables 1, 3), which could well explain the observed phenotypes in cell growth, migration, apoptosis, ROS and mitochondrial membrane potential in KO cells. Other GO and KEGG differences include morphogenesis and organ development, reflecting the established functions of hypoxia responses. DMOG treatment still induced the HIF-1 signaling pathway in KO cells (Table 2), confirming miR-210 is not essential for hypoxia responses. Together, these global gene expression analyses lend support to our phenotypic observations and provide clues to future mechanistic studies.

miRNAs act by inhibiting the expression of target genes, so to understand the mechanisms of miR-210, we sought to identify miR-210 target genes. There were 955 upregulated genes in KO cells compared to the WT, and 714 of them were predicted miR-210 targets by miRWalk (Figure 7A; Supplementary Table S3). This result suggests that most genes upregulated in our KO cells are the direct targets of miR-210. These genes were enriched in expected functions such as cell migration, regulation of cell proliferation, response to wounding, cancers and diabetes (Table 4). Such genes would be the prime candidates for future studying the regulatory mechanisms by miR-210. Nonetheless, of the 156 previously reported miR-210 targets, only 9 were present in our list of 955 genes, a surprisingly low number (Figure 7A; Supplementary Table S3). Most well-known targets, such as *HOXA1* and *ISCU*, did not increase expression in the KO 293T cells (Figure 7B). Thus, our unexpected results suggest that the relationship between human miR-210 and target genes requires revisiting, careful validation, and investigation at more physiologically relevant settings. As gene expression is subject to multiple layers of regulation, it is possible that compensatory changes might mask the effect of miR-210 loss in 293T cells. *miR-210* KO in mice have phenotypes in the immune and respiratory systems (Wang H. et al., 2014; Mok et al., 2013; White et al., 2015; Krawczynski et al., 2016; Bian et al., 2021; Watts et al., 2021). Our GO and KEGG analyses identified few changes related to immunity or lung functions (Table 1–4), also implicating miR-210 functions as context dependent.

miR-210 had been reported to both stimulate and inhibit cell proliferation (Rothé et al., 2011; Tsuchiya et al., 2011; Yoshino et al., 2017b). As a potential mechanism, miR-210 targets a number of cell cycle related genes (**INTRODUCTION**). The polyclonal loss of miR-210 in renal cell carcinoma cells increased tumor growth in a xenograft model, and while detailed cellular phenotype and gene expression studies were not performed, *TWIST1* was suggested as a contributing miR-210 target (Yoshino et al., 2017b). Yet most of those cell cycle genes including *TWIST1* did not increase their mRNA expression in our KO 293T cells (Supplementary Table S3). *miR-210* KO increased the mRNAs of *CDC25B* and *FAM83D* (Supplementary Table S3), but these genes advance mitosis, in conflict with our KO cells having fewer G2/M cells (Figure 3). RNA-seq data analyses suggested DNA replication being affected in KO cells (e.g., Tables 1, 2), which might contribute to some of the growth and cell cycle phenotypes. As a large number of genes are involved in the cell cycle control and are subject to regulation at multiple levels such as protein stability, subcellular localization, post-translation modification, and complex formation, how miR-210 regulates the cell cycle or whether its effect depends on specific cellular environments requires future studies. Thus, our current study examined the apoptosis phenotype first. We found elevated levels of BNIP3L, a pro-apoptotic factor, might explain partly why *miR-210* KO enhanced apoptosis, as changes in mitochondrial membrane potential and ROS could also be related to apoptosis. BNIP3L is stimulated by hypoxia (Bruick, 2000) but has not been reported as a miR-210 target, even though it was predicted by miRWalk (Sticht et al., 2018). We showed that *miR-210* KO consistently increased BNIP3L mRNA and protein expression, and that miR-210 was able to suppress BNIP3L expression directly (Figure 8). A model is proposed in Figure 8F: hypoxia stabilizes HIF-1α, which activates miR-210 and BNIP3L (and many other genes). BNIP3L induces apoptosis, but such an effect is attenuated by miR-210 inhibition of BNIP3L expression. In other words, miR-210 serves as a brake in the overall hypoxia-induced apoptosis response downstream of HIF-1α. We’d like to note that, as pathway analyses identified changes in apoptosis upon miR-210 KO (e.g., Table 3), BNIP3L is likely only one of the multiple genes targeted by miR-210 in apoptosis.

In summary, the current study constructed human *miR-210* KO in 293T cells and analyzed the cell phenotype and gene expression systematically. The results indicated that human miR-210 plays a protective role in cell growth and apoptosis. Superficially, it could mean that increased miR-210 expression in many cancers might promote tumor growth and survival. But it would be simplistic to conclude that miR-210 drives tumorigenesis, as miR-210 might also act as a brake in certain aspects of the hypoxia responses, and some cancers indeed express lower miR-210 (Khalilian et al., 2023). Whether the conclusions from 293T cells can be extrapolated to other cells including cancers and the better understanding of miR-210 target genes and functions will require further research in a context-dependent manner.

## Data Availability

The datasets presented in this study can be found in online repositories. The names of the repository/repositories and accession number(s) can be found in the article/Supplementary Material.
