# Supplementary material for: miR-210 loss leads to widespread phenotypic and gene expression changes in human 293T cells
Source: Front Genet. 2024 Dec 16;15:1486252. doi: 10.3389/fgene.2024.1486252 (PMC11683127; doi:10.3389/fgene.2024.1486252)
Supplement: Supplementary file 2 [file Table1.doc]

Supplementary Table 1. Information for off-target analyses. Letters in red represent mismatches to the miR-210 sgRNA.

| Potential matches to sgRNA | PAM | Score | Locus | Forward and reverse primers |
| --- | --- | --- | --- | --- |
| AGGGGCTGCATTGCGCCTGG | CAG | 2.67 | chr4: +41613727 | F: AGTGGCTACATCGACTGCTG |
|  |  |  |  | R: TCACTGAGTCCAAGGGAGGA |
| AGGGCCTGCCCTGCTCCTGG | TGG | 2.31 | chr15: -68209665 | F: CCCAGGCTAGGCGTTTTGTA |
|  |  |  |  | R: GCATTGTCACAGTCCCCACT |
| GAGGGCGGCCCTGCGCCTGG | GGG | 1.83 | chr1: +1779061 | F: GACATTGTGATCCCAGCCCG |
|  |  |  |  | R: CTACAAAGTCCGCTCCTGTGG |
| GGCGGCCGCCCTGCGCCTGG | AGG | 1.80 | chr10: +95183534 | F: CACTTCCTGGCGGACCTGAC |
|  |  |  |  | R: AGGACCTGGGCTTCCTGTCT |
| CGGGGCTGCCCTGAGCCTGG | CAG | 1.65 | chr1: -226827275 | F: GTTGTCTAGCTGCCGGAGTAT |
|  |  |  |  | R: TCCATGCAGGAATTCCACACG |
| GCGGGGTGCCCTGCGCCTGG | CAG | 1.56 | chr6: -157488815 | F: CCTCAAGGGTCGCTGGTTCAT |
|  |  |  |  | R: GATCTGCTGATGGAAGCCCG |
| GGAGGCTGCCCGGCGCCTGG | AGG | 1.56 | chr1: +25811851 | F: CCAAGTTCTGAATGGCAGCG |
|  |  |  |  | R: CCCTCTGCAATGAGTGGAGC |
| AGTGAGTGCCCTGCGCCTGG | GAG | 1.45 | chr16: -2263080 | F: CATCTTGCCCTCAGGTCTCG |
|  |  |  |  | R: GCTTAGGAGATGGGCTCCGC |
| AGCGGCTACCCAGCGCCTGG | CGG | 1.44 | chr15: -82043927 | F: CGAGAACGACTTCCACGCCA |
|  |  |  |  | R: GATAAGTGGTGCCCCAGGTG |
| ACGTCCTTCCCTGCGCCTGG | AGG | 1.44 | chr7: +150341593 | F: GGCTGGATCGAGGGGAAGGA |
|  |  |  |  | R: ACGACCCCTCTTCCTAGCCC |
| TGGGGCTGCCTTGCGCCTGT | GGG | 1.10 | chr14: +23522123 | F: TGACAGGCAGCACTGGCATC |
|  |  |  |  | R: CTCCCACCGAACACCCCTTG |
