# Supplementary material for: miR-210 loss leads to widespread phenotypic and gene expression changes in human 293T cells
Source: Front Genet. 2024 Dec 16;15:1486252. doi: 10.3389/fgene.2024.1486252 (PMC11683127; doi:10.3389/fgene.2024.1486252)
Supplement: Supplementary file 3 [file Table2.doc]

| Locus | Sanger sequencing for KO#1 | Sanger sequencing for KO#2 | Sanger sequencing for KO#3 |
| --- | --- | --- | --- |
| chr4: +41613727 | 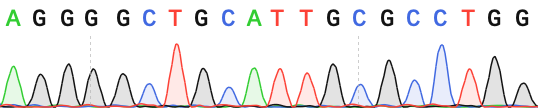 | 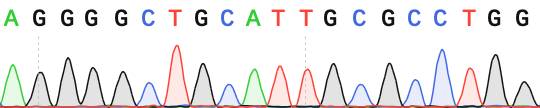 | 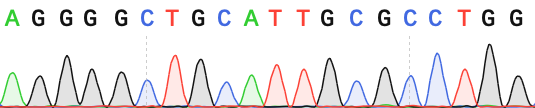 |
| chr15: -68209665 | 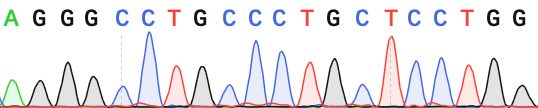 | 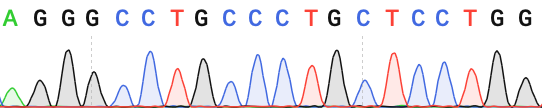 | 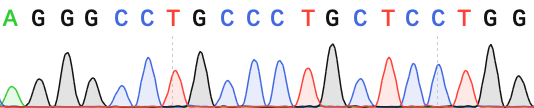 |
| chr1: +1779061 | 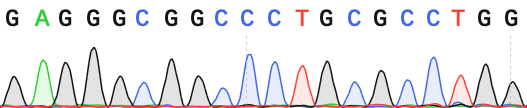 | 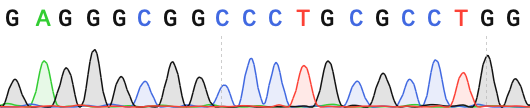 | 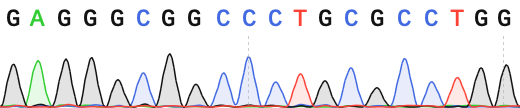 |
| chr10: +95183534 | 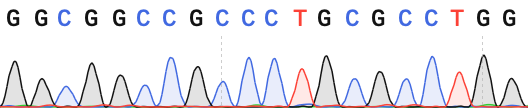 | 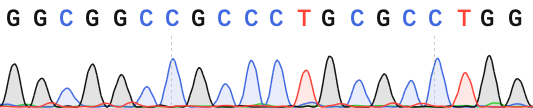 | 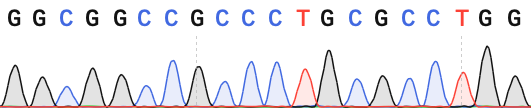 |
| chr1: -226827275 | 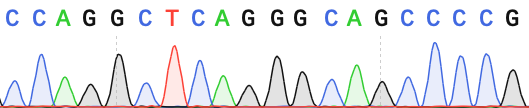 | 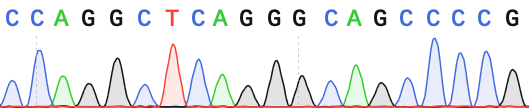 | 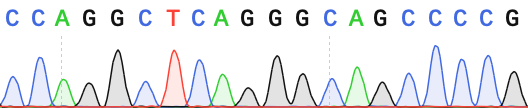 |
| chr6: -157488815 | 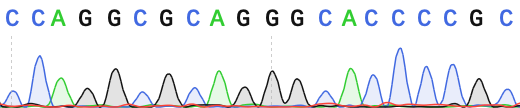 | 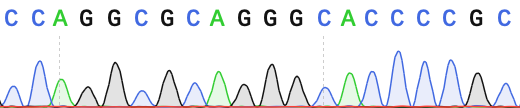 | 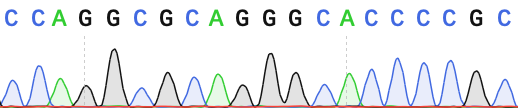 |
| chr1: +25811851 | 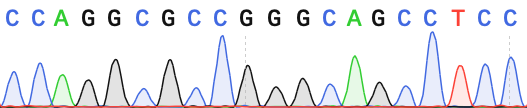 | 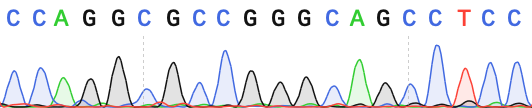 | 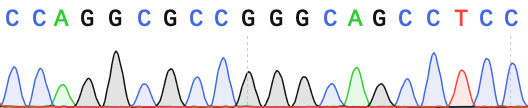 |
| chr16: -2263080 | 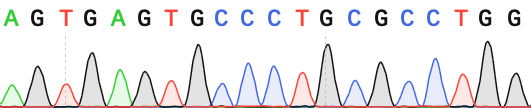 | 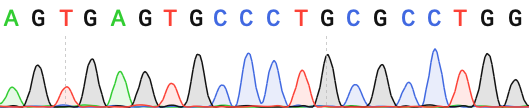 | 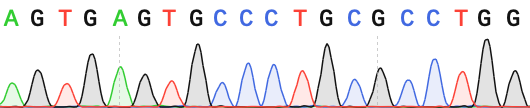 |
| chr15: -82043927 | 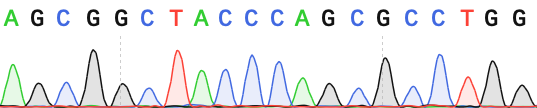 | 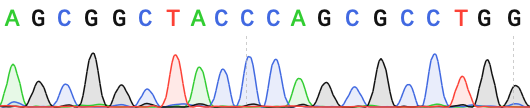 | 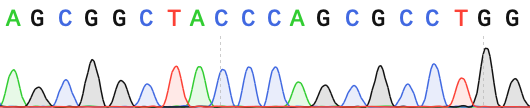 |
| chr7: +150341593 | 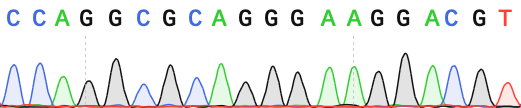 | 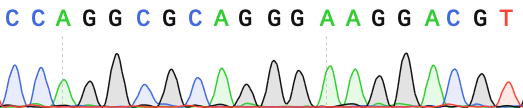 | 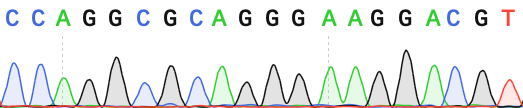 |
| chr14: +23522123 | 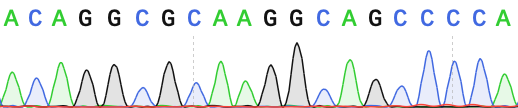 | 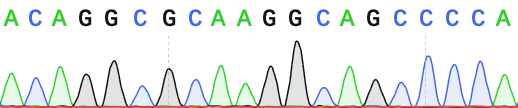 | 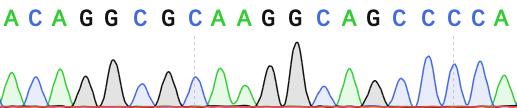 |

Supplementary Table 2. miR-210 sgRNA off-target site analyses in the KO#1, 2, and 3 cells. Examples of Sanger sequencing data are shown. No mutations were detected.
